# Modeling the ecology of parasitic plasmids

**DOI:** 10.1038/s41396-021-00954-6

**Published:** 2021-04-08

**Authors:** Jaime G. Lopez, Mohamed S. Donia, Ned S. Wingreen

**Affiliations:** 1 Lewis-Sigler Institute for Integrative Genomics, Princeton University, Princeton, NJ USA; 2 Department of Molecular Biology, Princeton University, Princeton, NJ USA

**Keywords:** Theoretical ecology, Microbial ecology

## Abstract

Plasmids are autonomous genetic elements that can be exchanged between microorganisms via horizontal gene transfer (HGT). Despite the central role they play in antibiotic resistance and modern biotechnology, our understanding of plasmids’ natural ecology is limited. Recent experiments have shown that plasmids can spread even when they are a burden to the cell, suggesting that natural plasmids may exist as parasites. Here, we use mathematical modeling to explore the ecology of such parasitic plasmids. We first develop models of single plasmids and find that a plasmid’s population dynamics and optimal infection strategy are strongly determined by the plasmid’s HGT mechanism. We then analyze models of co-infecting plasmids and show that parasitic plasmids are prone to a “tragedy of the commons” in which runaway plasmid invasion severely reduces host fitness. We propose that this tragedy of the commons is averted by selection between competing populations and demonstrate this effect in a metapopulation model. We derive predicted distributions of unique plasmid types in genomes—comparison to the distribution of plasmids in a collection of 17,725 genomes supports a model of parasitic plasmids with positive plasmid–plasmid interactions that ameliorate plasmid fitness costs or promote the invasion of new plasmids.

## Introduction

Plasmids are autonomous genetic elements that utilize the replication machinery of a host to replicate. They come in a variety of forms, ranging from plasmids of only a few kilobases that contain no discernible genes [1], to large, chromosome-like plasmids that encode genes essential to host survival [2]. Plasmids can transfer between hosts by a variety of mechanisms, including conjugation, by which cells directly exchange plasmids [3], and transformation, in which free plasmids infect cells [4]. Plasmids are important vehicles of horizontal gene transfer (HGT) in bacteria and archaea, being one of the mechanisms that allow these clonally reproducing organisms to share genetic information [5]. As such, plasmids play a key role in the dissemination of antibiotic resistance genes among pathogens [6, 7]. For example, one survey of *Salmonella enterica* genomes found that over 80% of the identified antibiotic resistance genes were contained within plasmids [8]. In addition to carrying and transmitting these genes, plasmids can influence gene persistence and evolution in subtle ways. Plasmids physically link different genes in a manner that promotes co-selection, potentially increasing the persistence of these genes [9]. Plasmids with multiple copies have been shown to accelerate the evolution of novel antibiotic resistance variants, allowing populations to survive environmental changes that would drive populations with only chromosomally-encoded resistance genes to extinction [10].

The impact of a plasmid on its host’s ability to respond to environmental factors is an important aspect of plasmid ecology, but plasmids are not simply genetic accessories. Plasmids have a complex ecology that is also influenced by different routes of plasmid transfer and by a plethora of plasmid–plasmid interactions [11, 12]. Despite the significant role plasmids play in evolution and public health, many aspects of their natural ecology are not well understood. Most notably, it has not yet been definitively established how plasmids are able to persist over evolutionary timescales and not simply be integrated into the chromosome to minimize replication costs. In addition to this existential question, the factors governing the distribution of plasmids in nature have yet to be elucidated. Within a single species, there can be a wide variation of plasmid numbers and it is not understood why some strains contain large numbers of plasmids while others contain none.

There are two major mechanisms that could allow plasmids to be maintained: (1) Positive selection—plasmids are beneficial such that plasmid-containing cells out-compete plasmid-free cells. Scenarios based on positive selection also often include additional mechanisms to explain why beneficial plasmids are not eventually integrated into the chromosome. (2) Infectious transfer—costly plasmids could be maintained if they spread fast enough to compensate for reduced host growth. We refer to a plasmid that requires infectious transfer to persist as an “infectious plasmid”. Hypotheses invoking positive selection have historically been dominant in the literature with many works, primarily relying on early measurements of conjugation rates [13, 14], asserting that natural HGT rates are too slow for infectious spread of plasmids [12, 15–17]. There is also some recent experimental evidence for positive selection being required for plasmid persistence in laboratory strains [18]. However, there is now a growing body of experimental evidence that HGT can indeed be fast enough to maintain costly plasmids. Early work by Lundquist demonstrated costly plasmids successfully invading plasmid-free populations [19], and recent work has shown plasmids spreading in the absence of positive selection in laboratory strains and natural hosts [20–22]. In many cases it is still difficult to determine whether a given natural plasmid is truly an infectious plasmid, owing to the fact that the functions of many plasmid-borne genes have yet to be understood. However, the aforementioned experimental results demonstrate that parasitism is a viable plasmid lifestyle. What are the ramifications of these findings for plasmid ecology?

Mathematical models have played an important role in understanding the results of plasmid experiments. Analysis of early models of conjugative plasmids yielded conditions for persistence that are widely utilized in interpreting experimental results [23]. Since this early work, mathematical models have been extended to study plasmid ecosystems beyond those that can be created in the laboratory. These ecological models of plasmids have primarily focused on the case of beneficial plasmids, in particular those carrying antibiotic resistance genes and genes enabling cooperative behaviors [16, 24–26]. There has been comparatively little theoretical work on parasitic infectious plasmids, with only a handful of papers exploring this scenario. These papers have generally focused on conjugative plasmids in single well-mixed environments [20, 27, 28], with exploration of more complex scenarios limited to generalized models of mobile genetic elements [29, 30].

Motivated by experimental evidence that natural plasmids can exist as infectious parasites, we ask: what are the implications for plasmid ecology? We begin with single plasmid, single species models and find that different modes of HGT can lead to qualitatively different infection strategies and population dynamics. We then model plasmid co-infection and find a plasmid “tragedy of the commons” in which runaway invasions by plasmids reduce the fitness of the host to arbitrarily low levels. We propose that the resolution of this plasmid runaway lies on a higher level of selection: in metapopulation models, plasmid invasions are limited by HGT barriers between populations. From a Wright–Fisher type model, we derive the predicted distribution of the number of unique plasmid types per genome and show that the form of the distribution varies depending on plasmid epistasis (i.e. plasmid–plasmid interactions that influence plasmid fitness costs or the invasion rate of new plasmids). We find that the observed distribution in a collection of 17,725 genomes is consistent with a model of parasitic plasmids with positive epistasis.

## Results

### Single plasmid, single-population models

**Fig. 1:**
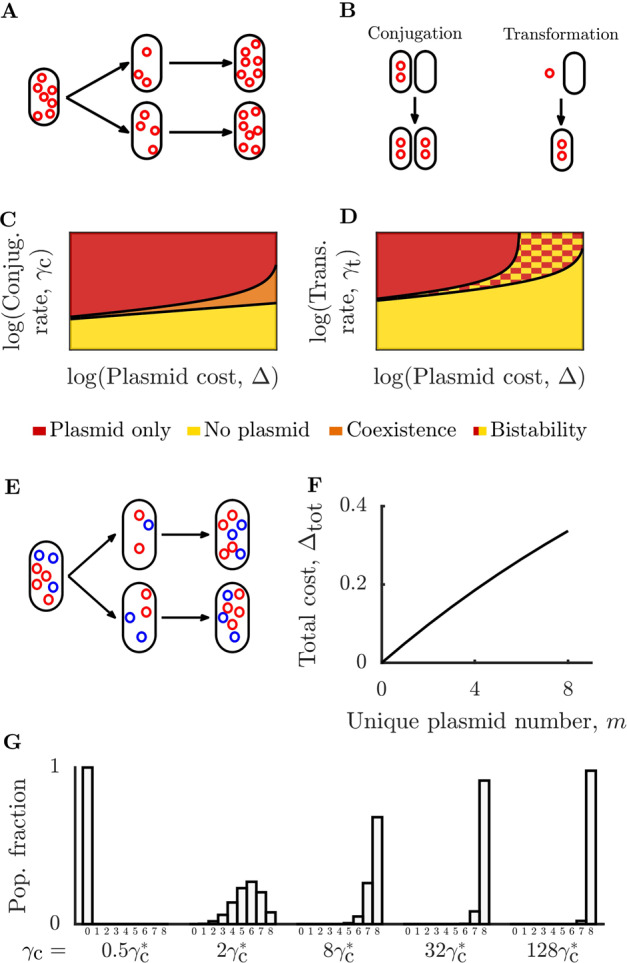
Different modeled mechanisms of plasmid transfer lead to distinct ecological phase diagrams, but all such mechanisms leave individual populations susceptible to runaway plasmid invasion. **A** At each division, plasmids are randomly segregated between daughter cells. Original plasmid copy number is regenerated if at least one plasmid remains in a daughter cell. **B** Schematic of plasmid transfer mechanisms. Left: spread of plasmids by plasmid-containing cells conjugating with plasmid-free cells. Right: spread of plasmids by extracellular plasmids infecting plasmid-free cells via transformation. **C** Phase diagram for conjugative plasmids as a function of plasmid cost, ${\Delta}$, and $\gamma _{\mathrm{c}}$; $\delta \,=\, 0.1$, $S \,=\, 1$, $p_\ell \,=\, 0$, and $\alpha \,=\, 1$ (see Eq. 4). **D** Phase diagram for transformative plasmids as a function of ${\Delta}$ and $\gamma _{\mathrm{t}}$. Parameters as in *C* with $\delta _{\mathrm{p}} \,=\, 0.3$ and $n_{{\mathrm{eff}}} \,=\, 0.6$ (see Eq. 9). See “Methods” for details. **E** In model multiplasmid cells, plasmid types segregate independently. If at least one plasmid of a given type remains in a daughter cell, the full copy number of that plasmid type is regenerated. **F** Fitness cost as a function of number of unique plasmid types in a cell for multiplicative case ${\Delta}_{{\mathrm{tot}}} \,=\, 1 \,-\, (1 \,-\, {\Delta})^m$ with ${\Delta} \,=\, 0.05$. **G** Steady-state distribution of number of plasmid types per cell at different conjugation rates, measured relative to $\gamma _{\mathrm{c}}^ \ast$ (the critical conjugation rate necessary for invasion of a single plasmid into a plasmid-free population, see Eq. 4). Results for eight unique plasmid types with $\delta \,=\, 1$, ${\Delta} \,=\, 0.1$, $\alpha \,=\, 1$, $S \,=\, 1$, and $p_\ell \,=\, 0.05$.

To understand the dynamics of parasitic plasmids in complex ecologies, we first need to understand their behavior in simple scenarios. In this section, we analyze the dynamics of plasmids spreading by different HGT mechanisms in single populations. We begin by modeling competition between plasmid-free cells and cells containing a conjugative plasmid. A nutrient, with concentration $C$, is supplied to the system at rate $S$. Cells grow at a rate proportional to $C$ with proportionality constant $\alpha$ for plasmid-free cells or $(1 \,-\, {\Delta})\alpha$ for plasmid-containing cells. Since we are interested in parasitic plasmids, we assume that ${\Delta} \in (0,1)$. Cells of both types die at a rate $\delta$. When a plasmid-containing cell divides there is a loss probability, $p_\ell$, for one of the daughter cells to contain no plasmids. As long as a daughter cell contains at least one plasmid, the original plasmid copy number (the number of copies of the plasmid maintained per cell) is regenerated (as depicted in Fig. 1A). Plasmids can spread horizontally by conjugation, as illustrated in Fig. 1B, wherein a plasmid-free cell and a plasmid-containing cell interact to produce two plasmid-containing cells. We model the rate of conjugation by a mass-action term with rate $\gamma _{\mathrm{c}}$. The equations governing the dynamics of conjugation are therefore:

1-3 $$	\frac{{d\rho }}{{dt}} \,=\, \alpha C\rho \,-\, \gamma _{\mathrm{c}}\rho \rho _{\mathrm{p}} \,+\, p_\ell (1 \,-\, {\Delta})\alpha C\rho _{\mathrm{p}} \,-\, \delta \rho ,\\ 	 \frac{{d\rho _{\mathrm{p}}}}{{dt}} \,=\, (1 \,-\, {\Delta})\alpha C\rho _{\mathrm{p}} \,+\, \gamma _{\mathrm{c}}\rho \rho _{\mathrm{p}} \,-\, p_\ell (1 \,-\, {\Delta})\alpha C\rho _{\mathrm{p}} \,-\, \delta \rho _{\mathrm{p}},\\ 	 \frac{{dC}}{{dt}} \,=\, S \,-\, \alpha C\rho \,-\, (1 \,-\, {\Delta})\alpha C\rho _{\mathrm{p}}.$$

In this model, what are the conditions for a parasitic conjugative plasmid to be able to invade a plasmid-free population? Invasibility implies that the equilibrium containing only plasmid-free cells is locally unstable, which occurs when

4 $$\qquad\qquad\qquad\gamma _{\mathrm{c}}\rho ^ \ast \,> \, \delta {\Delta} \,+\, \delta p_\ell (1 \,-\, {\Delta}),$$

where $\rho ^ \ast \,=\, S/\delta$ is the steady-state abundance of the plasmid-free cells at the plasmid-free equilibrium. This invasibility condition has an intuitive physical interpretation: to invade, the rate of conjugation must overcome losses due to reduced host growth rate as well as plasmid loss during division. This condition is similar to those found in previous studies [15].

Given the condition for plasmid invasion in Eq. 4, what is the optimal behavior for a parasitic conjugative plasmid? The left-hand-side of the expression is linear in the plasmid-free population, meaning that it is more difficult for a plasmid to invade smaller populations. To favor invasion, the plasmid can minimize the right-hand-side of the equation. For a plasmid that relies on random segregation upon cell division, both the plasmid cost ${\Delta}$ and the loss probability $p_\ell$ are functions of plasmid copy number, $n_{\mathrm{p}}$, a property controlled by the plasmid itself. If the primary cost of a plasmid is its replication and its gene products, plasmid cost will scale with copy number such that ${\Delta} \,=\, {\Delta}_{\mathrm{p}}n_{\mathrm{p}}$, where ${\Delta}_{\mathrm{p}}$ is the cost of an individual plasmid copy. The loss probability will be $p_\ell \,=\, 2^{1 \,-\, n_{\mathrm{p}}}$, i.e., the probability that a daughter cell receives zero plasmids from random segregation. The right-hand-side of the invasion condition Eq. 4 is therefore $\delta ({\Delta}_{\mathrm{p}}n_{\mathrm{p}} \,+\, 2^{1 \,-\, n_{\mathrm{p}}}(1 \,-\, {\Delta}_{\mathrm{p}}n_{\mathrm{p}}))$, which has a minimum at finite $n_{\mathrm{p}}$. The minimum in the invasion boundary at finite $n_{\mathrm{p}}$ indicates that in our framework optimal conjugative plasmids have a moderate copy number.

What kinds of ecological dynamics does our model for a conjugative parasitic plasmid exhibit? To answer this question, we characterize the stability of the system’s equilibria (see SI Appendix 1 for details). For conjugative plasmids with the optimal copy number, the dominant form of loss will be from reduced host fitness (see SI Fig. S1), and thus we characterize the case of negligible loss rate $p_\ell \,=\, 0$ (we consider the case of finite loss rates in SI Fig. S2 and find similar results). In Fig. 1C we show the phase diagram of possible ecological outcomes as a function of plasmid cost ${\Delta}$ and conjugation rate $\gamma _{\mathrm{c}}$. For high values of plasmid cost and low values of conjugation rate, the plasmid is unable to invade and the plasmid-free equilibrium is the only stable state. As plasmid cost decreases or conjugation rate increases, plasmids are able to invade and there is a state of stable coexistence between plasmid-free and plasmid-containing cells. The range of conjugation rates permitting coexistence is larger for costlier plasmids. Once the plasmid cost is sufficiently low or the conjugation rate is sufficiently high, the unique stable state consists only of plasmid-containing cells (note that for finite values of loss rate $p_\ell$, this plasmid-only state will contain a small fraction of plasmid-free cells due to plasmid loss).

Conjugation is the best studied mechanism of plasmid transmission, but plasmids can instead be transmitted by transformation, whereby plasmid-free cells are infected by free-floating plasmids, as illustrated in Fig. 1B. We therefore consider a model for plasmid-spread via transformation in which cell death results in release of free-floating plasmids which can then infect cells by mass action at rate $\gamma _{\mathrm{t}}$. For every cell death, $n_{{\mathrm{eff}}}$ free-floating plasmids are released and these plasmids decay at a rate $\delta _{\mathrm{p}}$. The dynamics of transformative plasmids are therefore:

5-8 $$	\frac{{d\rho }}{{dt}} \,=\, \alpha C\rho - \gamma _{\mathrm{t}}\rho P \,+\, p_\ell (1 \,-\, {\Delta})\alpha C\rho _{\mathrm{p}} \,-\, \delta \rho ,\\ 	\frac{{d\rho _{\mathrm{p}}}}{{dt}} \,=\, (1 \,-\, {\Delta})\alpha C\rho _{\mathrm{p}} \,+\, \gamma _{\mathrm{t}}\rho P \,-\, p_\ell (1 \,-\, {\Delta})\alpha C\rho _{\mathrm{p}} \,-\, \delta \rho _{\mathrm{p}},\\ 	\frac{{dC}}{{dt}} \,=\, S \,-\, \alpha C\rho \,-\, (1 \,-\, {\Delta})\alpha C\rho _{\mathrm{p}},\\ 	\frac{{dP}}{{dt}} \,=\, n_{{\mathrm{eff}}}\delta \rho _{\mathrm{p}} \,-\, \gamma _{\mathrm{t}}\rho P \,-\, \delta _{\mathrm{p}}P.$$

What is the condition for transformative plasmid invasion? The plasmid-free equilibrium is unstable if

9 $$\qquad\qquad\quad\gamma _{\mathrm{t}}\rho ^ \ast \,> \, \delta _{\mathrm{p}}\left( {\frac{{{\Delta} \,+\, p_\ell (1 \,-\, {\Delta})}}{{n_{{\mathrm{eff}}} \,-\, {\Delta} \,-\, p_\ell (1 \,-\, {\Delta})}}} \right).$$

The left-hand-side of Eq. 9 is similar to the conjugative plasmid invasion condition, with the conjugation rate $\gamma _{\mathrm{c}}$ replaced by the transformation rate $\gamma _{\mathrm{t}}$. The numerator of the right-hand-side is also similar, with the cell death rate $\delta$ replaced with the plasmid decay rate $\delta _{\mathrm{p}}$. The primary difference is in the denominator, which is the difference between the number of plasmids released on cell death, $n_{{\mathrm{eff}}}$, and the total replication deficit of plasmid-containing cells. If this denominator is negative, the inequality reverses and the plasmid-free equilibrium is always stable.

The invasion condition in Eq. 9 determines the optimal $n_{\mathrm{p}}$ of transformative plasmids: if each plasmid within a cell has a fixed probability of remaining viable after cell death, $p_{\mathrm{v}}$, then $n_{{\mathrm{eff}}}$ will scale linearly with $n_{\mathrm{p}}$ such that $n_{{\mathrm{eff}}} \,=\, p_{\mathrm{v}}n_{\mathrm{p}}$. If the denominator of Eq. 9 is positive, the optimal copy number will be $n_{\mathrm{p}} \,=\, 1/{\Delta}_{\mathrm{p}}$, the point at which the host’s growth rate is driven to zero and the plasmid relies entirely on horizontal transfer to survive. These results are substantially different than in the case of conjugation: instead of restricting itself to a limited portion of the host’s metabolic budget, a transformative parasite maximizes its spread by using as much of the host’s resources as possible. This is reminiscent of the behavior of phages—suggesting a possible evolutionary link between parasitic plasmids and phages.

As in the conjugation case, we now explore the ecological outcomes possible with transformative plasmids. We again consider the case of negligible loss rate $p_\ell \,=\, 0$ and characterize the stability of the equilibria (see SI Appendix 1 for details). For $n_{{\mathrm{eff}}} \,> \, 1$, the system has similar ecological outcomes to the conjugative case, with the system transitioning through no-plasmid, coexistence, and plasmid-only equilibria as ${\Delta}$ decreases and $\gamma _{\mathrm{t}}$ increases. Interestingly, when $n_{{\mathrm{eff}}} \,<\, 1$, there is now a regime in which both the plasmid-only and no-plasmid states are locally stable, leading to bistability (Fig. 1D). A community in this bistable regime is subject to dramatic composition shifts if subjected to sufficiently large perturbations.

Our simple models suggest that both conjugative and transformative parasitic plasmids can persist for sufficiently low fitness burdens and sufficiently high transfer rates. However, these different transfer mechanisms lead to significantly different optimal infection strategies and ecological outcomes. Conjugative plasmids in our framework have moderate copy optimal number, while transformative plasmids behave in a virus-like manner and produce as many copies as possible.

### Multiplasmid, single-population models

Whether by invasion from other populations or mutation of existing plasmids, a population of cells is likely to encounter a wide variety of plasmids. To explore this scenario, we expand our model to allow for co-infection by multiple parasitic plasmids. We assume plasmids are from different incompatibility groups, meaning they do not interfere with each other’s replication, segregation, or other functions necessary for plasmid persistence and can coexist over long times [31]. We show a schematic of independently segregating plasmids in Fig. 1E. We also assume that plasmids transfer between cells independently. As shown in Fig. 1F, fitness costs are assumed to be multiplicative, such that the total cost for $m$ plasmids is ${\Delta}_{{\mathrm{tot}}}(m) \,=\, 1 \,-\, (1 \,-\, {\Delta})^m$. Note that assuming additive fitness costs does not qualitatively change the following results, and for small plasmid costs the two schemes are equivalent. For a system of $m$ plasmids, this leads to $2^m \,+\, 1$ deterministic equations (we show an example model in SI Appendix 2). If the plasmids have identical properties and begin at equal abundances, this can be reduced to a system of $m \,+\, 2$ equations.

For the case of highly infectious plasmids, we can infer the behavior of the multiplasmid model from the single-plasmid dynamics. Consider a plasmid that is sufficiently infectious such that when it invades a population, every plasmid-free cell is infected. Now consider the case of another similarly infectious plasmid invading this population. Since every cell contains the first plasmid, the second plasmid has a similar (slightly smaller in the multiplicative case) effective fitness cost as it would have had invading a plasmid-free population. Thus, the second plasmid is also able to invade and infect all hosts. The second invasion deforms the fitness landscape further, meaning that a third plasmid can then invade. This process of repeated invasions will lead to plasmids reducing the host fitness to arbitrarily low levels. These runaway invasions can be viewed as a plasmid “tragedy of the commons”. In the classical tragedy of the commons, individuals acting in their own self-interest deplete a shared resource until it is unusable for all individuals. In this case, the host cell is a common resource for plasmids. Individual plasmids only use part of the cell’s resources, but together they can completely deplete the resource, ultimately destroying the host.

What about the case of moderately infectious plasmids that do not infect every plasmid-free cell? When multiple moderately infectious plasmids invade a population, the result is a distribution of cells with varying numbers of unique plasmid types per cell. If a single plasmid stably infects a fraction $p$ of the population, then if another identical plasmid invades and is completely independent of this first plasmid, it will also infect a fraction $p$ of the population. Thus, if plasmids are identical and independent, the plasmids will be distributed among cells as ${\mathrm{Binomial}}(m,p)$ where $m$ is the number of unique plasmid types. If plasmids are independent but not identical, they will be distributed as a Poisson binomial. While real plasmids are not independent of each other (they are coupled by their fitness costs to the host), the distribution of plasmids with low fitness cost will be well-approximated by a Poisson binomial. The discrepancy between the true distribution and a Poisson binomial will grow as fitness cost increases (see SI Fig. S3). In Fig. 1G, we show an example of this distribution for the case of eight identical moderately conjugative infectious plasmids. Below the critical conjugation rate for a single plasmid, $\gamma _{\mathrm{c}}^ \ast$, plasmids do not persist. As the conjugation rate increases past $\gamma _{\mathrm{c}}^ \ast$, the distribution of unique plasmid types per cell becomes increasingly skewed towards the total number of unique plasmid types. Since the distribution of plasmid types is binomial-like, as the total number of unique plasmids in the system increases, the mean number of unique plasmid types per cell increases as well (for the exact binomial this mean is $mp$, where $m$ is the number of unique plasmid types). Thus, even invasion by moderately infectious plasmids can lower host fitness to arbitrarily low levels. At the single-population level, there is no mechanism to stop this process, except perhaps extinction of the population, which may occur with finite population sizes (see SI Appendix for details). However, even if hosts with extremely high numbers of plasmids went extinct, this repeated invasion scenario would still imply that hosts with large numbers of unique plasmid types should be common in nature. In striking contrast to this scenario, prior analyses have found that most genomes contain only 0–3 plasmids [12]. What mechanisms might be limiting plasmid invasion in nature?

### Muliplasmid, multipopulation models

In single populations, runaway plasmid invasion occurs because each invading plasmids further deforms the fitness landscape. However, the natural world does not consist of a single well-mixed population. In nature, different populations are separated by physical and genetic barriers that limit HGT. These barriers limit the ability of plasmids to spread, possibly stopping runaway plasmid invasion.

**Fig. 2:**
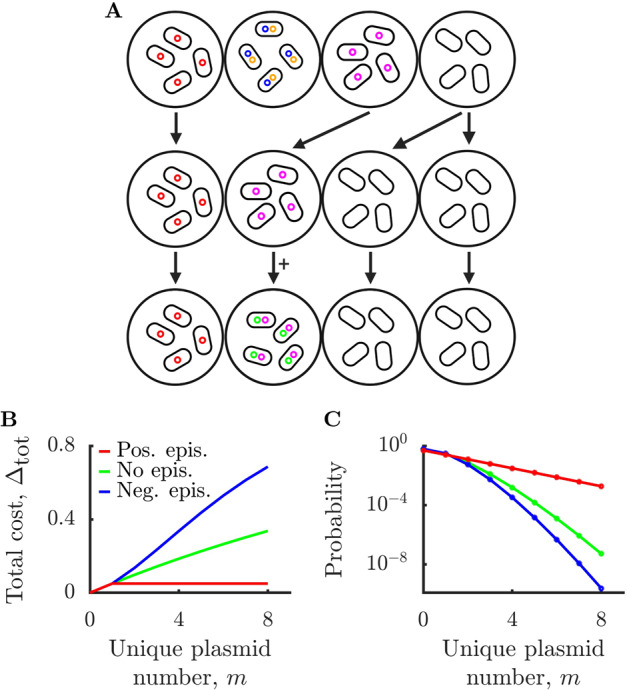
Competition between populations may prevent runaway plasmid invasion. **A** Illustration of multiple populations, each occupying an isolated “deme”. During each epoch, populations compete for demes, with plasmid invasion occurring randomly (see Eq. 11 for details). In the example shown, in the first epoch, the population with two plasmids is replaced by the population with zero plasmids. In the second epoch, the population with magenta plasmids is invaded by the green plasmid. **B** Multiplasmid fitness costs for different types of epistasis. With no epistasis, fitness burden is multiplicative as in Fig. 1F. With positive epistasis, fitness burden increases sub-multiplicatively (pictured: ${\Delta}_{{\mathrm{tot}}} \,=\, {\Delta}$ for $m \,> \, 0$). For negative epistasis, fitness burden increases super-multiplicatively (pictured: ${\Delta}_{{\mathrm{tot}}} \,=\, 1 \,-\, (1 \,-\, {\Delta})^{m^{3/2}}$). **C** Steady-state distributions of number of plasmid types per cell in the Wright–Fisher model (see SI Appendix 3). Parameters ${\Delta} \,=\, 0.01$ and plasmid invasion probability for each time period $q \,=\, 0.005$.

To explore the impact of HGT barriers, we use a Wright–Fisher framework to model a metapopulation of cells. We model a set of $N$ populations living in $N$ isolated demes. At every time period (“epoch”), a new resident of each deme is selected from a weighted distribution of the current $N$ populations. The probability of being selected is proportional to the average fitness of the population, with a population containing $i$ plasmids having fitness of $w_i$. During every epoch, each population may also be invaded by a new plasmid type with probability $q$. We assume the invading plasmid is compatible with the existing plasmids within the cell (we explore case of significant incompatibility interactions in the SI Appendix). We assume that adaptation of an invading plasmid to a new population occurs on a timescale much faster than the length of a epoch, such that successful adaptation is accounted for within the invasion probability. We illustrate three epochs of this process in Fig. 2A. Formally, each epoch can be described as $N$ draws from the following multinomial distribution:

10-11 $$\begin{array}{*{20}{c}} \quad{p_i \,=\, \frac{{n_0w_0(1 \,-\, q)}}{{\mathop {\sum }\nolimits_{j \,=\, 0}^\infty n_jw_j}}} & {i \,=\, 0,} \\ \qquad\qquad\quad{p_i \,=\, \frac{{n_iw_i(1 \,-\, q) \,+\, n_{i \,-\, 1}w_{i \,-\, 1}q}}{{\mathop {\sum }\nolimits_{j \,=\, 0}^\infty n_jw_j}}} & {i \,> \, 0.} \end{array}$$

A population’s fitness is dependent on the number of unique plasmid types it contains. Thus far, we have considered a simple multiplicative model. However, it has been demonstrated that plasmid–plasmid interactions can modulate plasmid properties. For example, one study found that the presence of a plasmid can reduce the fitness cost of an invading plasmid [12]. To account for this epistasis between plasmids, we also consider fitness costs that increase sub-multiplicatively (positive epistasis) or super-multiplicatively (negative epistasis). We show examples of positive epistasis, negative epistasis, and no epistasis in Fig. 2B.

What is the distribution of unique plasmid types across populations in our model with HGT barriers? We derive the stationary distribution of this model for the three different epistasis functions in Fig. 2B and plot them in Fig. 2C (see SI Appendix 3 for details). For the case of no epistasis, the stationary distribution is Poisson-like. Positive epistasis favors carriage of multiple plasmids and results in an exponential-like distribution with a long tail. Negative epistasis has the opposite effect: it penalizes carriage of multiple plasmids and results in a sub-Poissonian distribution with a reduced tail. Importantly, in all cases the runaway invasion of plasmids is stopped. While there is nothing stopping individual populations from being overrun by invading plasmids, these populations are more likely to be out-competed by populations with fewer plasmids. Thus, the single-population “tragedy of the commons” is counteracted at a higher level of selection.

### Analysis of natural genomes

**Fig. 3:**
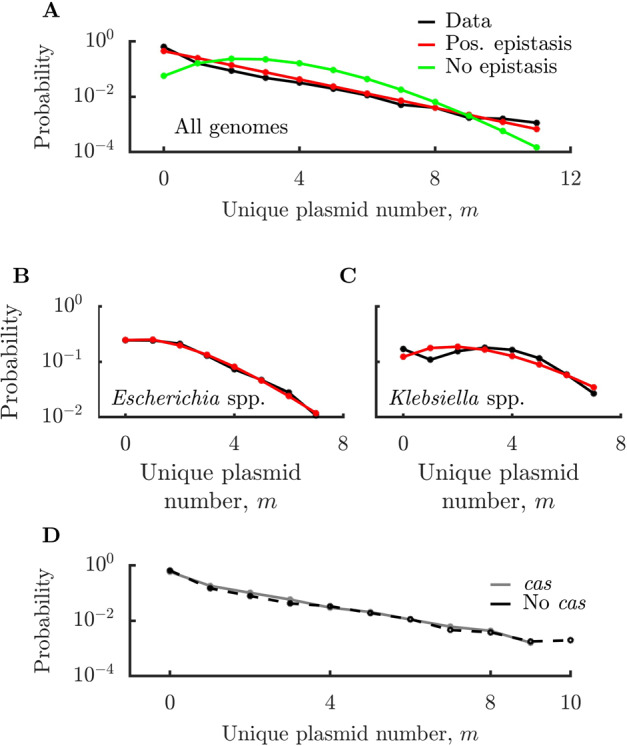
Comparison of distributions of number of unique plasmid types per cell in natural genomes to Wright–Fisher model. **A** Distribution of number of plasmid types per cell in 17,725 complete NCBI genomes. Positive epistasis distribution fit with the fitness function ${\Delta}_{{\mathrm{tot}}} \,=\, {\Delta}$ for $m \,> \, 0$ (best-fit parameters: ${\Delta} \,=\, 9.8 \,\times\, 10^{ - 3}$, $q \,=\, 5.4 \,\times\, 10^{ - 3}$), no epistasis distribution fit with ${\Delta}_{{\mathrm{tot}}} \,=\, 1 \,-\, (1 \,-\, {\Delta})^m$ (best-fit parameters: ${\Delta} \,=\, 3.9 \,\times\, 10^{ - 3}$, $q \,=\, 1.4 \,\times\, 10^{ - 2}$). **B** Distribution of number of plasmid types per cell in 1153 complete *Escherichia* genomes, with a positive epistasis fit using the fitness function ${\Delta}_{{\mathrm{tot}}} \,=\, 1 \,-\, (1 \,-\, {\Delta})^{m^a}$ (best-fit parameters: ${\Delta} \,=\, 8.3 \,\times\, 10^{ - 3}$, $q \,=\, 8 \,\times\, 10^{ - 3}$, $a \,=\, 0.33$). **C** Distribution of number of plasmid types per cell in 576 complete *Klebsiella* genomes, with a positive epistasis fit using the fitness function as in (**B**) (best-fit parameters: ${\Delta} \,=\, 7 \,\times\, 10^{ - 3}$, $q \,=\, 9.7 \,\times\, 10^{ - 3}$, $a \,=\, 0.43$). Note that in certain limits of our models, only the ratio of $q$ and ${\Delta}$ can be properly estimated, effectively reducing them to single parameter (see SI Appendix 3). **D** Distribution of number of plasmid types per cell in genomes containing and not containing *cas* genes. Genomes are considered *cas* containing if at least one chromosome or plasmid within the genome contains a *cas* gene. See “Methods” for details.

How does our predicted distribution of unique plasmid types per cell compare to that in natural genomes? To make this comparison, we downloaded all complete bacterial genomes from NCBI (a total of 17,725 genomes) and analyzed their plasmid content. In Fig. 3A, we show the overall distribution of unique plasmid types per genome and corresponding model fits for both positive and no epistasis cases (see “Methods” for fitting details). The natural distribution is exponential-like and is well-fit by a model with positive epistasis. The model fit with no epistasis has too short a tail to be able to fit the data, and this problem becomes even more severe for negative epistasis. Thus, interestingly, we find that the distribution of unique plasmid types in real-world genomes is consistent with parasitic plasmids that ameliorate each other’s fitness costs. The degree of positive epistasis suggested by the data is quite strong—the distribution is nearly a pure exponential. In our model, this corresponds to the case in which the cost of all plasmids beyond the first is zero, such that for $m \,> \, 1$ the parameters controlling both population replication and plasmid invasion are independent of plasmid number. This means that the ratio between consecutive elements of the distribution is constant, yielding an exponential tail. In order to determine whether our conclusions are influenced by oversampling of clinically relevant species, we excluded 91 genera known to be clinically relevant or human-associated and repeated our analysis. The remaining dataset contains nearly 5000 genomes and still shows clear exponential behavior (see SI Fig. S4). We also analyzed whether the presence of engineered strains within the NCBI database influences our results. We found that there are only a small number of these engineered strains and that removing them had negligible impact on our results (see SI Fig. S5).

Can our model capture variation within smaller, related groups of genomes? In Fig. 3B we show the distribution of unique plasmid types per cell within the genus *Escherichia*. As can be seen, the data is very well fit by a model of parasitic plasmids with positive epistasis. However, our model was not able to capture some of the within-genus distributions we encountered. A notable exception is the distribution of unique plasmid types per cell in the genus *Klebsiella*, shown in Fig. 3C. In this genus, there is a substantial discontinuity between the zero-plasmid class and the rest of the distribution. While our simple Wright–Fisher model with some positive epistasis can capture the tail of the distribution, it then fails to capture the first few classes. Despite such exceptions, we find that the positive epistasis model is generally able to capture the overall trends in plasmid distributions over the bulk of natural genomes (see SI Fig. S6).

It should be noted that our current model of constant plasmid invasion probability and strong positive epistasis is not the only Wright–Fisher model that can produce an exponential distribution matching the data. We analyzed a more general form of the Wright–Fisher model in which the invasion probability and total fitness cost are arbitrary functions of unique plasmid number (see SI Appendix). We find that the general condition to yield an exponential is that the plasmid invasion probability and total fitness cost must be comparable regardless of the number of plasmids in the cell. These results indicate that even if there is no epistasis in fitness cost, an exponential can still result if there is positive epistasis in the invasion probability (i.e., if existing plasmids make it more likely for a new plasmid to successfully invade).

HGT barriers are not the only mechanism that can plausibly limit runaway plasmid invasion. Cells also have specialized systems to defend against foreign DNA, notably the CRISPR-Cas system [32]. To explore whether CRISPR-Cas is responsible for limiting plasmid invasion in natural genomes, we searched for *cas* genes within the NCBI complete bacterial genomes using HMMER (see “Methods” for details). We expect that if CRISPR-Cas plays a major role in limiting the spread of plasmids, the distribution of unique plasmid types per cell would be shifted towards lower plasmid numbers in genomes containing *cas* genes versus those lacking *cas* genes. In Fig. 3D, we show the distribution of unique plasmid types per genome in genomes containing at least one *cas* gene and those not containing any *cas* genes. The distributions are very similar, with no large differences between them. These results suggest that CRISPR-Cas is not a major mechanism limiting the spread of plasmids in bacteria. There are additional defense systems that may also influence plasmid carriage. However, a prior bioinformatics study found results similar to ours for restriction-modification (RM) systems, another defense system that protects against foreign DNA; the study examined the distribution of RM systems in bacterial genomes and found almost no relationship between the number of RM systems a genome encodes and the presence of plasmids (in one subset of data the authors actually found a positive relation) [33].

## Discussion

Plasmids play a significant role in bacterial evolution and the spread of antibiotic resistance, but their ecology is not well understood. Inspired by experiments demonstrating that plasmids can exist parasitically, we used simple mathematical models to explore the implications of these findings for the distribution of plasmids in nature. By analyzing models across multiple population scales, we developed a mechanistic framework to provide insight into the forces shaping the natural ecology of plasmids.

Our single-plasmid models revealed that plasmid ecology is strongly dependent on the mechanism of HGT. Our analysis predicts that a conjugative plasmid maximizes its ability to invade populations by having moderate copy number and consuming only a modest fraction of the host’s budget, in-line with previous explorations of the trade-off between segregation loss and host burden [34]. This appears to reflect the reality of conjugative plasmids as they typically have low copy numbers [5] and a moderate fitness cost ${\Delta}$ [15, 35]. While we specifically considered the case of plasmids that rely on random segregation, this conclusion should hold for other segregation mechanisms as well. If a plasmid uses an active segregation mechanism, its optimal copy number will likely be lower than a plasmid that relies on random segregation. Interestingly, our model of transformative plasmids leads to a very different outcome. Unlike a conjugative plasmid, a plasmid relying on transformation can enhance its transmission by increasing its copy number. This means that a transformative plasmid’s optimal infection strategy is to behave in a phage-like manner and produce as many copies as possible. Thus far, there is no direct experimental evidence for the existence of parasitic transformative plasmids, though there are natural plasmids with high copy numbers in-line with those expected from our model ($n_{\mathrm{p}} \,\sim\, 10^2$) [36]. Presently, the possibility of transformative parasitic plasmids cannot be ruled out as there has been relatively little experimental work studying the ecology of plasmids spread by natural transformation.

While we explicitly modeled conjugative and transformative plasmids, our framework is useful for understanding the ecological and evolutionary implications of other transfer mechanisms. We predict that HGT mechanisms that are enhanced by copy number promote phage-like behavior, while those not enhanced by copy number will lead to more restrained parasites. For example, it was recently shown that plasmids can use specialized vesicles to spread between cells [37]. If higher copy number allows for the production of more plasmid vesicles, the plasmid’s optimal behavior will likely be phage-like, similar to a transformative plasmid. Interestingly, plasmids can also use phage to transfer between cells via a mechanism known as transduction [38]. Since this mechanism involves extracellular vectors, our results indicate that the large burst sizes phage use to propagate are also optimal for plasmid spread. Our single-plasmid results can also be useful in understanding the behavior of non-plasmid parasitic genetic elements. For example, viroids are pathogenic circular RNA elements encoding no known protein. They are plant pathogens transmitted by leaf-to-leaf contact or contaminated tools [39], a mechanism that likely benefits from an increased number of copies. As expected from our framework, viroids place a severe, sometimes lethal, burden on their hosts [39].

Our analyses of multiple plasmids in a single-population highlight the broader ecological pressures faced by parasitic plasmids. Without additional limiting mechanisms, plasmids in our model are prone to a “tragedy of the commons” within a single population. Due to the relative nature of fitness, existing plasmids do not prevent the spread of new plasmids, leading to runaway invasions. We find that the distribution of plasmid types within a population depends on the strength of the transfer mechanism and the cost of the plasmids, with the most extreme scenario being one in which all cells contain all plasmids.

While we limited our multiplasmid analyses to plasmids from different incompatibility groups, competition of plasmids in the same incompatibility group can lead to additional forms of selection pressure. If two incompatible plasmids share a copy-control mechanism, it may be beneficial for a plasmid to increase its copy number to out-compete the other plasmid [34]. Even in this case, there will be substantial differences between plasmids with different transfer mechanisms. For a conjugative plasmid, this intra-host pressure will conflict with inter-host pressure to maintain a moderate copy number, while for a transformative plasmid the intra-host pressure will align with the inter-host pressure to increase copy number. This additional pressure towards higher copy numbers will likely worsen the tragedy of the commons. Indeed, a tragedy of the commons between incompatible plasmids has been observed in experiments [40].

We suggest that the tragedy of the commons between parasitic plasmids is counteracted by competition between populations isolated by HGT barriers. Correspondingly, within a Wright–Fisher model, we are able to provide a dynamic explanation for the distribution of plasmids in nature. We find that real distributions are consistent with strong positive epistasis between plasmids, a phenomenon that has been observed in experiments [12]. Interestingly, while our model is able to fit the plasmid distributions within many genera, some distributions contained sharp variations that our model cannot account for. This suggests the presence of additional factors governing plasmid acquisition and loss, such as environment-specific parameters. Our model of a balance between parasite invasion and selection is conceptually similar to those developed for transposons [41], and there are generalized models of mobile genetic elements that rely on a similar mechanism [30]. However, a major difference is that these models are concerned with the distribution of copy numbers, while we model the distribution of plasmid types.

In addition to HGT barriers, we explored CRISPR-Cas as a possible mechanism limiting plasmid transfer. Somewhat surprisingly, we found no substantial difference in the distribution of unique plasmid types for cells harboring *cas* genes and those not harboring *cas* genes. This is counterintuitive, as it has been shown experimentally that a CRISPR-Cas system can prevent plasmid invasion [32]. However, our findings are consistent with a recent bioinformatics study finding no negative relationship between the rate of HGT and CRISPR-Cas activity [42]. A potential explanation for these results is that the presence or absence of CRISPR-Cas systems may change on a rapid timescale, such that genomes are intermittently protected by CRISPR-Cas, but not all genomes have them at a given time. This discrepancy may also be partially explained by limitations of our bioinformatic analyses. We focused on CRISPR-Cas systems as they are well-studied and widespread, but other, possibly undiscovered, defense systems may also play a role in limiting plasmid invasion. That said, a previous study found results similar to ours for RM systems, another major bacterial defense system [33].

We took a primarily ecological view in this work, but understanding plasmid evolution is equally important. Parasitic plasmids are now known to exist, but what drives their evolution? Our multiplasmid models raise interesting questions about the evolution of plasmid interactions. For example, our analysis of natural plasmid distributions suggests that plasmids generally mitigate each other’s fitness costs, but why would parasites competing for a host cooperate? We hope that our modeling can serve as a foundation for further theory and experiments characterizing plasmid ecology and evolution.

## Methods

For analysis of the single-plasmid models, phase diagrams were constructed using linear stability analysis of equilibria (see SI Appendix 1). For multiplasmid models, steady-state distributions of plasmids were found by numeric simulation of model equations using a fourth-order Runge-Kutta method. For the Wright–Fisher model, the form of the steady-state distribution of number of unique plasmid types per genome was found analytically (see SI Appendix 3).

For bioinformatic analyses, all complete bacterial genomes (as of April 2020) were downloaded from NCBI. To account for non-complete assemblies mistakenly labeled as complete, genomes were excluded from the analysis if they did not contain a chromosome with length $$> $$0.5 Mb. The number of unique plasmid types in a genome was taken as the number of sequences labeled “Plasmid” in the NCBI assembly summary. For identification of *cas* genes, genomes were translated using Transeq into all six possible reading frames. The translated genomes were searched using hmmsearch against the database of *cas* HMMs from CRISPR CasFinder [43]. Identification of a *cas* gene was considered valid if the *E*-value of the match is below $$10^{ - 30}$$. For fitting of model distributions to data, distributions were truncated to only entries with at least ten observations. Distributions were fit by minimizing the sum of squared errors between the logarithms of the probability values. The MATLAB function lsqnonlin was used for the fitting process. All code used in this paper can be found at https://github.com/jaimegelopez/ParasiticPlasmidEcology.

## Supplementary information

Supplemental material
