# Publisher Correction: Modeling the ecology of parasitic plasmids

**DOI:** 10.1038/s41396-021-00997-9

**Published:** 2021-06-30

**Authors:** Jaime G. Lopez, Mohamed S. Donia, Ned S. Wingreen

**Affiliations:** 1 Lewis-Sigler Institute for Integrative Genomics, Princeton University, Princeton, NJ USA; 2 Department of Molecular Biology, Princeton University, Princeton, NJ USA

**Keywords:** Theoretical ecology, Microbial ecology

Correction to: *The ISME Journal*

10.1038/s41396-021-00954-6

Owing to errors in production, the original version of this article unfortunately contained mistakes. The equations were not properly labeled:

1 $$\frac{{d\rho }}{{dt}} = \alpha C\rho - \gamma _{\mathrm{c}}\rho \rho _{\mathrm{p}} + p_\ell (1 - \Delta )\alpha C\rho _{\mathrm{p}} - \delta \rho,$$

2 $$\frac{{d\rho _{\mathrm{p}}}}{{dt}} = (1 - \Delta )\alpha C\rho _{\mathrm{p}} + \gamma _{\mathrm{c}}\rho \rho _{\mathrm{p}} - p_\ell (1 - \Delta )\alpha C\rho _{\mathrm{p}} - \delta \rho _{\mathrm{p}},$$

3 $$\frac{{dC}}{{dt}} = S - \alpha C\rho - (1 - \Delta )\alpha C\rho _{\mathrm{p}}.$$

4 $$\gamma _{\mathrm{c}}\rho ^ \ast \,> \, \delta \Delta + \delta p_\ell (1 - \Delta ),$$

5 $$\frac{{d\rho }}{{dt}} = \alpha C\rho - \gamma _{\mathrm{t}}\rho P + p_\ell (1 - \Delta )\alpha C\rho _{\mathrm{p}} - \delta \rho ,$$

6 $$\frac{{d\rho _{\mathrm{p}}}}{{dt}} = (1 - \Delta )\alpha C\rho _{\mathrm{p}} + \gamma _{\mathrm{t}}\rho P - p_\ell (1 - \Delta )\alpha C\rho _{\mathrm{p}} - \delta \rho _{\mathrm{p}},$$

7 $$\frac{{dC}}{{dt}} = S - \alpha C\rho - (1 - \Delta )\alpha C\rho _{\mathrm{p}},$$

8 $$\frac{{dP}}{{dt}} = n_{{\mathrm{eff}}}\delta \rho _{\mathrm{p}} - \gamma _{\mathrm{t}}\rho P - \delta _{\mathrm{p}}P.$$

9 $$\gamma _{\mathrm{t}}\rho ^ \ast \,> \, \delta _{\mathrm{p}}\left( {\frac{{\Delta + p_\ell (1 - \Delta )}}{{n_{{\mathrm{eff}}} - \Delta - p_\ell (1 - \Delta )}}} \right).$$

10 $$p_i = \frac{{n_0w_0(1 - q)}}{{\mathop {\sum }\nolimits_{j = 0}^\infty n_jw_j}}\quad \quad i = 0,$$

11 $$p_i = \frac{{n_iw_i(1 - q) + n_{i - 1}w_{i - 1}q}}{{\mathop {\sum }\nolimits_{j = 0}^\infty n_jw_j}}\quad \quad i \,> \, 0.$$

The original article has been corrected.

