# Supplementary material for: Modeling the ecology of parasitic plasmids
Source: ISME J. 2021 Apr 8;15(10):2843–52. doi: 10.1038/s41396-021-00954-6 (PMC8443676; doi:10.1038/s41396-021-00954-6)
Supplement: Supplementary file 1 — Supplemental material [file 41396_2021_954_MOESM1_ESM.pdf]

Supplemental material for "Modeling the ecology of parasitic plasmids"

Jaime G. Lopez[a], Mohamed S. Donia[b], and Ned S. Wingreen[a,b]

[a]Lewis-Sigler Institute for Integrative Genomics, Princeton University, New Jersey 08544, USA

[b]Department of Molecular Biology, Princeton University, New Jersey 08544, USA

February 24, 2021

Contents

1 Single plasmid, single population ODE models 3

1.1 Conjugative plasmids . . . 3

1.1.1 Model equations . . . 3

1.1.2 Condition for plasmid invasion . . . 3

1.1.3 Conjugative plasmid phase diagram . . . 4

1.2 Transformative plasmids . . . 6

1.2.1 Model equations . . . 6

1.2.2 Condition for plasmid invasion . . . 6

1.2.3 Transformative plasmid phase diagram . . . 7

1.3 Relating Δ, n_{eff}, and p_ℓ, to plasmid copy number . . . 9

1.4 Conjugation and transformation phase diagrams with finite loss rate p_ℓ . . . 9

1.5 Plasmid-driven extinction . . . 10

2 Multiplasmid, single population ODE models 11

3 Multiplasmid, multipopulation Wright-Fisher models **16**

3.1 Model formulation and stationary distribution 16

3.1.1 Case of positive epistasis 18

3.1.2 Case of arbitrary invasion and fitness costs 21

3.1.3 Incompatibility interactions 22

1 Single plasmid, single population ODE models

1.1 Conjugative plasmids

1.1.1 Model equations

The equations governing the dynamics of conjugative plasmids are:

$$\frac{d\rho}{dt} = \alpha C\rho - \gamma_{\mathrm{c}}\rho\rho_{\mathrm{p}} - \delta\rho + p_\ell(1-\Delta)\alpha C\rho_{\mathrm{p}}, \tag{S1}$$

$$\frac{d\rho_{\mathrm{p}}}{dt} = (1-\Delta)\alpha C\rho_{\mathrm{p}} + \gamma_{\mathrm{c}}\rho\rho_{\mathrm{p}} - \delta\rho_{\mathrm{p}} - p_\ell(1-\Delta)\alpha C\rho_{\mathrm{p}}, \tag{S2}$$

$$\frac{dC}{dt} = S - \alpha C\rho - (1-\Delta)\alpha C\rho_{\mathrm{p}}. \tag{S3}$$

where α is the growth rate of plasmid-free cells, ρ is the number density of plasmid-free cells, C is the nutrient concentration, γ_{c} is the conjugation rate, ρ_{p} is the number density of plasmid-containing cells, δ is the death rate of cells, p_ℓ is the plasmid loss coefficient, Δ is the plasmid fitness cost, and S is the nutrient supply rate.

1.1.2 Condition for plasmid invasion

It can be seen that there is a plasmid-free equilibrium, y_{f}^*, at

$$y_{\mathrm{f}}^* = \begin{bmatrix} \rho^* \\ \rho_{\mathrm{p}}^* \\ C^* \end{bmatrix} = \begin{bmatrix} S/\delta \\ 0 \\ \delta/\alpha \end{bmatrix}. \tag{S4}$$

If this equilibrium is locally unstable, plasmids can invade. The plasmid-free equilibrium will be unstable when the real part of the eigenvalues of the Jacobian evaluated at this equilibrium are greater than zero. In these eigenvalue calculations, we will take advantage of the fact that the eigenvalues of block upper or lower triangular matrices are the eigenvalues of the diagonal block matrices. To obtain a block lower triangular Jacobian, J_{f}, we reorder the equations to be $[\rho_{\mathrm{p}}, C, \rho]$, yielding

$$J_{\mathrm{f}} = \begin{bmatrix} (1-\Delta)\delta + \frac{\gamma_{\mathrm{c}}S}{\delta} - \delta - \delta p_\ell(1-\Delta) & 0 & 0 \\ -\delta(1-\Delta) & -\frac{S\alpha}{\delta} & -\delta \\ -\frac{\gamma_{\mathrm{c}}S}{\delta} + \delta(1-\Delta)p_\ell & \frac{S\alpha}{\delta} & 0 \end{bmatrix}. \tag{S5}$$

The submatrix in the lower right corner will always have negative eigenvalues since its $Tr = -\frac{S\alpha}{\delta} < 0$ and its $Det = S\alpha > 0$. Therefore plasmids will invade if

$$(1-\Delta)\delta + \frac{\gamma_c S}{\delta} - \delta - \delta p_\ell (1-\Delta) > 0. \tag{S6}$$

We can rearrange this as

$$\gamma_c \rho^* > \delta\Delta + \delta p_\ell (1-\Delta). \tag{S7}$$

This inequality has an intuitive physical interpretation: plasmid invasion requires the rate of conjugation to overcome the losses due to lower fitness and random plasmid loss events during division.

1.1.3 Conjugative plasmid phase diagram

To obtain the phase diagram of possible ecologically stable outcomes, we assume that the rate of plasmid loss is negligible such that $p_\ell = 0$. This system contains three equilibria: a no-plasmid equilibrium, a plasmid-only equilibrium, and an equilibrium where the two cell types coexist. We construct the phase diagram by assessing the conditions for local stability of all equilibria. The stability conditions of the no-plasmid and plasmid-only equilibria are explicitly derived here, while the stability of the coexistence equilibrium is determined by numerical analysis of the Jacobian in MATLAB. We have the plasmid-free equilibrium from Eq. S4, and with $p_\ell = 0$ there is now a plasmid-only equilibrium located at

$$y_s^* = \begin{bmatrix} \rho^* \\ \rho_p^* \\ C^* \end{bmatrix} = \begin{bmatrix} 0 \\ S/\delta \\ \delta/(1-\Delta)\alpha \end{bmatrix}. \tag{S8}$$

We already have the condition for the stability of the plasmid-free equilibrium (Eq. S7) which is $\gamma_c \rho^* < \delta\Delta$ when $p_\ell = 0$. The Jacobian at the plasmid-only equilibrium is

$$J_p = \begin{bmatrix} 0 & \frac{S(1-\Delta)\alpha}{\delta} & \frac{\gamma_c S}{\delta} \\ -\delta & -\frac{S(1-\Delta)\alpha}{\delta} & -\frac{\delta}{1-\Delta} \\ 0 & 0 & \frac{\delta}{1-\Delta} - \frac{\gamma_c S}{\delta} - \delta \end{bmatrix}. \tag{S9}$$

Note that this matrix is computed using the same order of the variables as in Eq. S5. The submatrix in the upper left will always have negative eigenvalues since it has $Tr = -\frac{S(1-\Delta)\alpha}{\delta} < 0$ and $Det = S(1-\Delta)\alpha > 0$.

Thus, the plasmid-only equilibrium is stable if

$$\frac{\delta}{1-\Delta} - \frac{\gamma_{\mathrm{c}} S}{\delta} - \delta < 0. \tag{S10}$$

We can rearrange this to

$$\gamma_{\mathrm{c}} \rho_{\mathrm{p}}^{*} > \left(\frac{1}{1-\Delta} - 1 \right) \delta. \tag{S11}$$

This has a similar interpretation to Eq. S7. In order to resist invasion by plasmid-free cells, the plasmid-containing cells must conjugate faster than $\left(\frac{1}{1-\Delta} - 1\right)\delta$, a rate that increases monotonically with fitness burden. When the fitness burden is small, this function is the same as in the Eq. S7: $\left(\frac{1}{1-\Delta} - 1\right)\delta = (\Delta + O(\Delta^2))\delta$, meaning that the two stability conditions converge as $\Delta \to 0$. Now that we have determined the conditions for stability of the pure strain equilibria, we can search for regions of coexistence or bistability within the phase diagram. Coexistence between plasmid-containing and plasmid-free cells will occur if both of the single-strain equilibria are unstable. This implies a region in the phase diagram where the conjugation rate is high enough for plasmids to invade the plasmid-free equilibrium, but not high enough for the plasmid-containing cells to resist invasion by the plasmid-free cells. Formally, this means that the critical conjugation rate for stability of the plasmid-free equilibrium is lower than the critical conjugation rate for stability of the plasmid-only equilibrium:

$$\left(\frac{1}{1-\Delta} - 1 \right) \frac{\delta}{\rho_{\mathrm{p}}^{*}} > \frac{\delta \Delta}{\rho^{*}}, \tag{S12}$$

$$\implies \Delta > 0. \tag{S13}$$

For parasitic plasmids, there will always be a region of the phase diagram where coexistence occurs. The condition for bistability between the plasmid-free and plasmid-only equilibria is the opposite of the coexistence condition, as it requires the plasmid-free critical conjugation rate be above the plasmid-only critical conjugation rate. Thus, for parasitic plasmids this system cannot support bistability between the plasmid-free and plasmid-only equilibria when $p_{\ell} = 0$. Note that even if $\Delta < 0$, the system cannot support bistability since Eq. S12 is true for all $\Delta \neq 0$.

1.2 Transformative plasmids

1.2.1 Model equations

The equations governing the dynamics of transformative plasmids are:

$$\frac{d\rho}{dt} = \alpha C\rho - \gamma_{\mathrm{t}}\rho P - \delta\rho + p_\ell(1-\Delta)\alpha C\rho_{\mathrm{p}}, \tag{S14}$$

$$\frac{d\rho_{\mathrm{p}}}{dt} = (1-\Delta)\alpha C\rho_{\mathrm{p}} + \gamma_{\mathrm{t}}\rho P - \delta\rho_{\mathrm{p}} - p_\ell(1-\Delta)\alpha C\rho_{\mathrm{p}}, \tag{S15}$$

$$\frac{dC}{dt} = S - \alpha C\rho - (1-\Delta)\alpha C\rho_{\mathrm{p}}, \tag{S16}$$

$$\frac{dP}{dt} = n_{\mathrm{eff}}\delta\rho_{\mathrm{p}} - \gamma_{\mathrm{t}}\rho P - \delta_{\mathrm{p}}P, \tag{S17}$$

where γ_t is the transformation rate, P is the number density of free plasmids, n_{eff} is the average number of plasmids released upon the death of a plasmid-containing cell, and δ_{p} is the decay rate of free plasmids.

1.2.2 Condition for plasmid invasion

We once again have a plasmid-free equilibrium, y_{f}^*, this time at

$$y_{\mathrm{f}}^* = \begin{bmatrix} \rho^* \\ \rho_{\mathrm{p}}^* \\ C^* \\ P^* \end{bmatrix} = \begin{bmatrix} S/\delta \\ 0 \\ \delta/\alpha \\ 0 \end{bmatrix}. \tag{S18}$$

Plasmids will invade if this equilibrium is unstable. We reorder the variables to be $[\rho, C, \rho_{\mathrm{p}}, P]$ to obtain J_{f}, a block triangular Jacobian at the plasmid-free equilibrium:

$$J_{\mathrm{f}} = \begin{bmatrix} 0 & \frac{\alpha S}{\delta} & \delta p_\ell(1-\Delta) & \frac{-\gamma_{\mathrm{t}}S}{\delta} \\ -\delta & -\frac{\alpha S}{\delta} & -(1-\Delta)\delta & 0 \\ 0 & 0 & (1-\Delta)\delta - \delta - \delta p_\ell(1-\Delta) & \frac{\gamma_{\mathrm{t}}S}{\delta} \\ 0 & 0 & n_{\mathrm{eff}}\delta & -\frac{\gamma_{\mathrm{t}}S}{\delta} - \delta_{\mathrm{p}} \end{bmatrix}. \tag{S19}$$

The submatrix in the upper left will always have negative eigenvalues since it has $Tr = -\frac{S\alpha}{\delta} < 0$ and $Det = S\alpha > 0$. Thus stability depends on the eigenvalues of the lower right submatrix. When the plasmid is costly, i.e. $(1-\Delta) < 1$, this matrix always has at least one negative eigenvalue because it has $Tr =$

$-\Delta\delta - \delta p_\ell(1-\Delta) - \frac{\gamma_t S}{\delta} - \delta_p < 0$. Thus plasmids will successfully invade if $Det < 0$, i.e. if

$$\frac{S\gamma_t}{\delta} > \delta_p \left(\frac{\Delta + p_\ell(1-\Delta)}{n_{eff} - \Delta - p_\ell(1-\Delta)} \right). \tag{S20}$$

Note that this inequality flips if $n_{eff} - \Delta - p_\ell(1-\Delta) < 0$. For a high-copy-number plasmid that releases many plasmids upon death ($n_{eff} \gg 1$), the plasmid invasion condition reduces to

$$\gamma_t \rho^* > \left(\frac{\delta_p}{n_{eff}} \right) (\Delta + p_\ell(1-\Delta)). \tag{S21}$$

This has a similar interpretation to the earlier invasion condition. For plasmid invasion, transformation rates must overcome losses due to fitness cost and random losses during cell division.

1.2.3 Transformative plasmid phase diagram

Similar to our analysis of the conjugation model, we build a phase diagram by analyzing the stability of the equilibria. We again assume that the plasmid loss rate is negligible ($p_\ell = 0$). This system also has plasmid-only, plasmid-free, and coexistence equilibria. We will explicitly derive the stability condition for the two pure strain equilibria here and assess the stability of the coexistence equilibrium by numerically analyzing the Jacobian in MATLAB. The stability condition for the plasmid-free equilibrium with $p_\ell = 0$ is

$$\frac{S\gamma_t}{\delta} < \frac{\delta_p \Delta}{n_{eff} - \Delta}. \tag{S22}$$

Also note that if the denominator is negative ($n_{eff} < \Delta$), the inequality flips, and because $\gamma_t > 0$, the plasmid-free equilibrium will always be stable. Next, we find the plasmid-only equilibrium to be:

$$y_p^* = \begin{bmatrix} \rho^* \\ \rho_p^* \\ c^* \\ P^* \end{bmatrix} = \begin{bmatrix} 0 \\ S/\delta \\ \delta/(1-\Delta)\alpha \\ n_{eff} S/\delta_p \end{bmatrix}. \tag{S23}$$

The Jacobian for this equilibrium (using the original order of the variables) is

$$J_{\text{p}} = \begin{bmatrix} \frac{\delta}{(1-\Delta)} - \frac{\gamma_{\text{t}} n_{\text{eff}} S}{\delta_{\text{p}}} - \delta & 0 & 0 & 0 \\ \frac{\gamma_{\text{t}} n_{\text{eff}} S}{\delta_{\text{p}}} & 0 & \frac{(1-\Delta)\alpha S}{\delta} & 0 \\ -\frac{\delta}{(1-\Delta)} & -\delta & -\frac{(1-\Delta)\alpha S}{\delta} & 0 \\ -\frac{\gamma_{\text{t}} n_{\text{eff}} S}{\delta_{\text{p}}} & n_{\text{eff}}\delta & 0 & -\delta_{\text{p}} \end{bmatrix}. \tag{S24}$$

J_{p} is block lower triangular, and the middle submatrix is the same as the matrix in the upper left of Eq. S9 and has only negative eigenvalues. Thus, the plasmid-only equilibrium is stable if

$$\frac{\delta}{1-\Delta} - \frac{\gamma_{\text{t}} n_{\text{eff}} S}{\delta_{\text{p}}} - \delta < 0. \tag{S25}$$

This can be rearranged to

$$\frac{\gamma_{\text{t}} n_{\text{eff}} S}{\delta_{\text{p}}} > \left(\frac{1}{1-\Delta} - 1\right)\delta. \tag{S26}$$

Note that the LHS is equal to $\gamma_{\text{t}} P^*$. This is similar in form to Eq. S11. For a population of plasmid-only cells to resist invasion by plasmid-free cells, the rate of transformation must overcome a rate that is monotonically increasing with plasmid cost. Using these stability conditions, we can explore whether this system can support coexistence or bistability. We begin with the condition for coexistence: the critical conjugation rate for stability of the plasmid-only equilibrium must be greater than that of the plasmid-free equilibrium, such that

$$\left(\frac{1}{1-\Delta} - 1\right)\frac{\delta\delta_{\text{p}}}{S n_{\text{eff}}} > \left(\frac{\delta_{\text{p}}\Delta}{n_{\text{eff}} - \Delta}\right)\frac{\delta}{S}. \tag{S27}$$

When $\Delta > 0$ and $n_{\text{eff}} > \Delta$, this reduces to

$$n_{\text{eff}} > 1. \tag{S28}$$

Thus, when these conditions are met and $n_{\text{eff}} > 1$, coexistence is possible. Like in the conjugation case, the condition for bistability between the plasmid-free and plasmid-only equilibria is the opposite of the condition for coexistence. Thus, when $n_{\text{eff}} < 1$, the phase diagram contains a region of bistability. Even if $n_{\text{eff}} < \Delta$ (only possible when $n_{\text{eff}} < 1$), the system will not support coexistence because the inequality in Eq. S22 will flip and the plasmid-free equilibrium will always be stable.

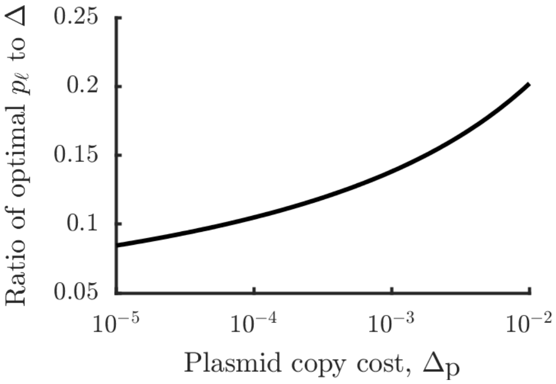

Fig. S1: Ratio of optimal segregation loss p_ℓ to optimal fitness cost Δ for different plasmid copy costs Δ_p. Using the same parameters as in Fig. 1C, we compute the n_p that minimizes the right-hand side of Eq. 4 and find the corresponding ratio of the optimal p_ℓ to Δ.

1.3 Relating Δ, n_{eff}, and p_ℓ, to plasmid copy number

Many of the parameters in the ODE models (see Eqs. S1-S3 and S14-S17) are strongly influenced by a single property: the copy number of the plasmid, n_p. Plasmid copy number is property that plasmids can themselves control by modulating their replication rate and is a major factor in plasmid evolution. How might plasmid cost Δ depend on n_p? If the cost of a plasmid primarily arises from its replication, it is reasonable to assume that the cost will scale with copy number, such that $\Delta = \Delta_\mathrm{p} n_\mathrm{p}$ where Δ_p is the cost per individual plasmid copy. The number of plasmids released upon cell death is also likely to scale with n_p, such that $n_{\text{eff}} = \epsilon n_p$ where ϵ is the fraction of plasmids that remain viable after cell death. How might the plasmid loss coefficient p_ℓ relate to copy number? If we assume that daughter cells receive plasmids from the parent cell by random segregation, it follows that $p_\ell = 2^{1-n_\mathrm{p}}$, i.e. the probability that one daughter cell receives zero plasmids.

As we discuss in the main text, the relationships between the model parameters and plasmid copy number allow us to compute optimal copy numbers that maximize the invasion potential of the plasmids. For a conjugative plasmid, this generally results in an intermediate optimal copy number. In Fig. S1 we characterize the relationship between the fitness cost Δ and segregation loss rate p_ℓ of optimal plasmids for different values of plasmid copy cost Δ_p. As can be seen, optimal conjugative plasmids have $p_\ell < \Delta$.

1.4 Conjugation and transformation phase diagrams with finite loss rate p_ℓ

In earlier sections we analytically characterized the stable ecological states of the single-plasmid models assuming negligible segregation loss rates ($p_\ell = 0$). To confirm that allowing $p_\ell > 0$ does not significantly alter the results, we numerically characterized the stable ecological states with finite segregation loss rates. In the

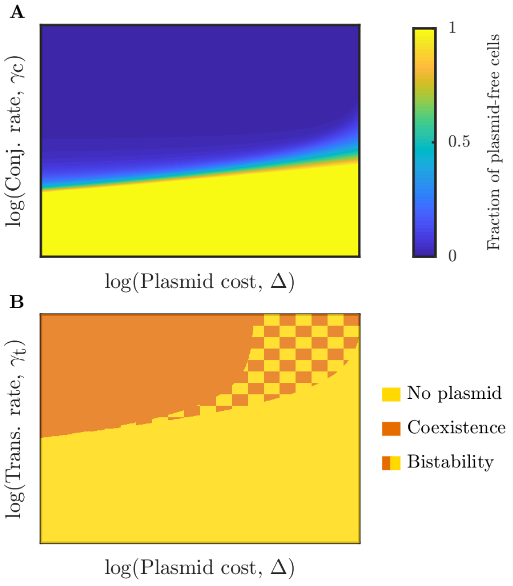

Fig. S2: Ecological outcomes for conjugation and transformation single-plasmid models with the same parameters as in Fig. 1, except with finite segregation loss rate $p_\ell = 0.05$. Stability of equilibria is determined by numerical analysis of the Jacobian. (*A*) Stable equilibrium fraction of plasmid-free cells as a function of conjugation rate γ_c and plasmid cost Δ. (*B*) Phase diagram for transformative plasmids with a finite loss rate.

case of conjugation, there is no longer a definite transition between plasmid-only and coexistence states. Since plasmid loss is finite, all equilibria contain at least a small fraction of plasmid-free cells. As such, instead of a phase diagram, we show a heatmap of the steady-state fraction of plasmid-free cells in Fig. S2A. As can be seen, there is still a transition between plasmid-containing and coexistence equilibria, but no true plasmid-only state. However, the behavior is qualitatively similar to that of the $p_\ell = 0$ as the plasmid-free cells become a negligible fraction of the population as plasmid cost decreases or conjugation rate increases. For transformation with finite loss rates, we show a phase diagram in Fig. S2B. As can be seen, bistability still occurs but is now between a plasmid-free state and a coexistence state.

1.5 Plasmid-driven extinction

Can a sufficiently virulent and costly plasmid drive its population to extinction? In order to answer this question we must modify the model to include a nutrient depletion term, as this will allow the existence of a equilibrium where no cells persist. We show the analysis for the conjugation model in detail here, but these results hold for the transformation model as well. First, we must modify Eq. S3 to be:

$$\frac{dC}{dt} = S - \alpha C \rho - (1-\Delta)\alpha C \rho_{\mathrm{p}} - \delta_{\mathrm{ca}} C. \tag{S29}$$

The extinction equilibrium of the system with therefore be:

$$y_{\text{e}}^* = \begin{bmatrix} \rho_{\text{p}}^* \\ C^* \\ \rho^* \end{bmatrix} = \begin{bmatrix} 0 \\ S/\delta_{\text{ca}} \\ 0 \end{bmatrix}. \tag{S30}$$

The corresponding Jacobian is:

$$J_{\text{e}} = \begin{bmatrix} -\delta + \frac{\alpha(1-p_\ell)(1-\Delta)S}{\delta_{\text{ca}}} & 0 & 0 \\ -\frac{\alpha(1-\Delta)S}{\delta_{\text{ca}}} & -\delta_{\text{ca}} & -\frac{\alpha S}{\delta_{\text{ca}}} \\ \frac{\alpha p_\ell(1-\Delta)S}{\delta_{\text{ca}}} & 0 & -\delta + \frac{\alpha S}{\delta_{\text{ca}}} \end{bmatrix}. \tag{S31}$$

This matrix is block triangular, and thus the eigenvalues are the eigenvalues of the diagonal blocks. By inspection, it can be seen that the largest eigenvalue is $-\delta + \frac{\alpha S}{\delta_{\text{ca}}}$. This eigenvalue is positive when plasmid-free cells are able to persist in the absence of plasmids. In the case of transformation, the eigenvalues of the extinction Jacobian are identical except for an additional negative eigenvalue corresponding to the decay rate of free plasmids. Thus, in biologically-relevant scenarios where cells persist in the absence of the plasmid, the extinction equilibrium is unstable. This means that plasmids in our model cannot drive the population to extinction. However, this result depends on populations being measured as continuous variables. With finite populations it is possible that a highly infectious plasmid could reduce the plasmid-free population to zero cells.

2 Multiplasmid, single population ODE models

For multiple plasmids in a single population, the models consist of $2^m + 1$ equations governing the dynamics of all possible types of plasmid-containing cell. For large m, these equations are difficult to represent and we instead developed a script to automatically generate and simulate these equations. As an example, we show the equations for $m = 2$:

$$\frac{d\rho}{dt} = \alpha C\rho - \gamma_c\rho(\rho_{10} + \rho_{01} + 2\rho_{11}) + (1-\Delta)\alpha C p_\ell(\rho_{10} + \rho_{01}) - \delta\rho, \tag{S32}$$

$$\frac{d\rho_{10}}{dt} = (1-\Delta)\alpha C\rho_{10} + \gamma_c\rho(\rho_{11} + \rho_{10}) - \gamma_c\rho_{10}(\rho_{01} + \rho_{11}) - (1-\Delta)\alpha C p_\ell\rho_{10} + (1-\Delta)^2\alpha C p_\ell\rho_{11} - \delta\rho_{10}, \tag{S33}$$

$$\frac{d\rho_{01}}{dt} = (1-\Delta)\alpha C\rho_{01} + \gamma_c\rho(\rho_{11} + \rho_{01}) - \gamma_c\rho_{01}(\rho_{10} + \rho_{11}) - (1-\Delta)\alpha C p_\ell\rho_{01} + (1-\Delta)^2\alpha C p_\ell\rho_{11} - \delta\rho_{01}, \tag{S34}$$

$$\frac{d\rho_{11}}{dt} = (1-\Delta)^2\alpha C\rho_{11} + \gamma_c(\rho_{11}\rho_{01} + \rho_{11}\rho_{10} + 2\rho_{10}\rho_{01}) - 2(1-\Delta)^2\alpha C p_\ell\rho_{11} - \delta\rho_{11}, \tag{S35}$$

$$\frac{dC}{dt} = S - \alpha C\rho - (1-\Delta)\alpha C\rho_{10} - (1-\Delta)\alpha C\rho_{01} - (1-\Delta)^2\alpha C\rho_{11}. \tag{S36}$$

The subscripts of ρ indicate plasmid content (e.g. ρ_{10} contains plasmid one but not plasmid two). If all plasmids are identical and begin at identical abundances, these equations can be condensed into a set of $m+2$ equations:

$$\frac{d\rho}{dt} = \alpha C\rho - \gamma_c\rho(\rho_1 + 2\rho_2) + (1-\Delta)\alpha C p_\ell\rho_1 - \delta\rho, \tag{S37}$$

$$\frac{d\rho_1}{dt} = (1-\Delta)\alpha C\rho_1 + \gamma_c\rho(2\rho_2 + \rho_1) - 2\gamma_c\left(\frac{\rho_1}{2}\right)^2 - \gamma_c\rho_1\rho_2 - (1-\Delta)\alpha C p_\ell\rho_1 + 2(1-\Delta)^2\alpha C p_\ell\rho_2 - \delta\rho_1, \tag{S38}$$

$$\frac{d\rho_2}{dt} = (1-\Delta)^2\alpha C\rho_2 + \gamma_c\rho_2\rho_1 + 2\gamma_c\left(\frac{\rho_1}{2}\right)^2 - 2(1-\Delta)^2\alpha C p_\ell\rho_2 - \delta\rho_2, \tag{S39}$$

$$\frac{dC}{dt} = S - \alpha C\rho - (1-\Delta)\alpha C\rho_1 - (1-\Delta)^2\alpha C\rho_2. \tag{S40}$$

Here, the subscript of ρ indicates number of unique plasmids (e.g. ρ_m has m unique plasmids). As $\Delta \to 0$, the steady-state distribution of plasmids among cells will approach a binomial (in the case of identical plasmids) or a Poisson binomial (in the case of non-identical plasmids). To demonstrate this, we show in Fig. S3 the difference between the binomial approximation and true solution for varying values of Δ in a system of three

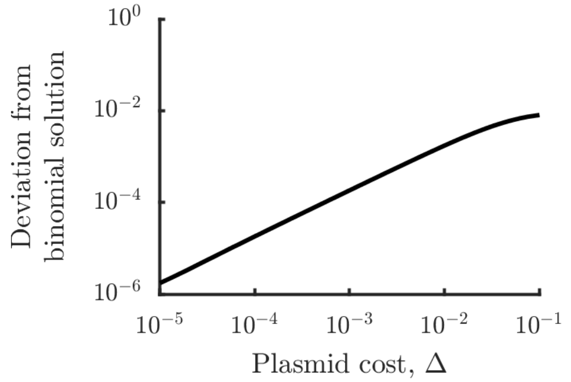

Fig. S3: Deviation from binomial approximation as a function of plasmid cost Δ for a system of three identical plasmids with the same parameters as in Fig. 2. For the binomial approximation, we analytically compute the steady-state fraction of plasmid-containing cells in a system containing a single plasmid and use this as the p in the binomial distribution. For each value of Δ, the conjugation rate is adjusted such that $\gamma_{\mathrm{c}} = 3\gamma_{\mathrm{c}}^{*}$, where γ_{c}^{*} is the critical conjugation rate needed for plasmid invasion. The solution to the full set of equations is found by numerical solution as in Fig. 2. The reported deviation is the 2-norm of the difference between the two plasmid distributions.

identical plasmids. As can be seen, the error approaches zero as Δ decreases.

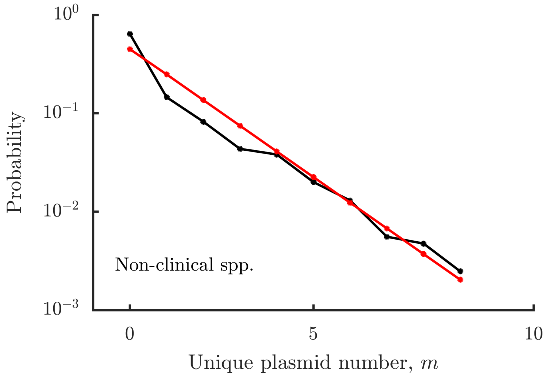

Fig. S4: Distributions of unique plasmid types per genome in non-clinically relevant genera, along with corresponding model fits. We excluded 91 clinically relevant genera from our initial set of 17 725 genomes, including all major pathogens and human commensal genera. The final dataset was composed of 4845 genomes. Distributions truncated and fit using the same method as in Fig. 4B and C, with fitting parameters $q = 0.0054$, $\Delta = 0.0098$. The genera removed were *Achromobacter*, *Acinetobacter*, *Actinobacillus*, *Actinomyces*, *Aeromonas*, *Akkermansia*, *Alcaligenes*, *Alistipes*, *Arcobacter*, *Bacillus*, *Bacteroides*, *Bartonella*, *Bifidobacterium*, *Blautia*, *Bordetella*, *Borrelia*, *Borreliella*, *Brucella*, *Burkholderia*, *Campylobacter*, *Candidatus*, *Capnocytophaga*, *Chlamydia*, *Chryseobacterium*, *Citrobacter*, *Clostridioides*, *Clostridium*, *Collinsella*, *Corynebacterium*, *Coxiella*, *Cronobacter*, *Cutibacterium*, *Desulfovibrio*, *Edwardsiella*, *Eggerthella*, *Elizabethkingia*, *Enterobacter*, *Enterococcus*, *Escherichia*, *Eubacterium*, *Faecalibacterium*, *Flavobacterium*, *Francisella*, *Fusobacterium*, *Gardnerella*, *Glaesserella*, *Haemophilus*, *Helicobacter*, *Histophilus*, *Hungateiclostridium*, *Klebsiella*, *Lactobacillus*, *Lactococcus*, *Leclercia*, *Legionella*, *Leptospira*, *Leuconostoc*, *Listeria*, *Microbacterium*, *Micrococcus*, *Moraxella*, *Mycobacterium*, *Mycobacteroides*, *Mycoplasma*, *Myroides*, *Neisseria*, *Nocardia*, *Pandoraea*, *Pantoea*, *Pasteurella*, *Prevotella*, *Propionibacterium*, *Proteus*, *Providencia*, *Pseudomonas*, *Rickettsia*, *Rothia*, *Salmonella*, *Serratia*, *Shigella*, *Sphingomonas*, *Staphylococcus*, *Stenotrophomonas*, *Streptococcus*, *Treponema*, *Ureaplasma*, *Veillonella*, *Vibrio*, and *Yersinia*. In certain rare cases where only a genome's family was annotated, we removed the families Enterobacteriaceae and Lachnospiraceae.

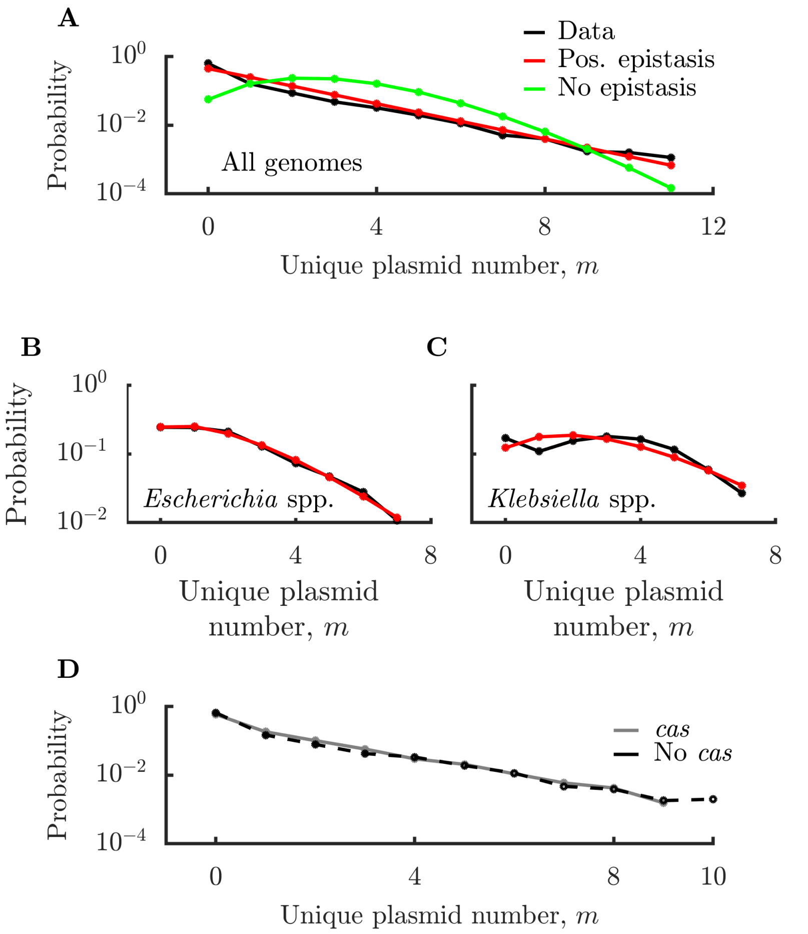

Fig. S5: Plasmid distribution analysis with putative engineered strains removed. The NCBI complete genomes dataset may contain genomes that have been artificially engineered, potentially skewing the observed plasmid distribution. To determine the impact of such genomes on our analysis, we filtered genomes based on the description of their associated Bioproject. We removed BioProjects containing the following strings in their description: 'engineer', 'clone', 'cloni', 'artificial', 'laboratory', 'recomb', 'industr', 'chimera', and 'Evolution of bacterial fitness with an expanded genetic code'. This filtering removed 173 genomes, and we then repeated the analyses found in Fig. 3. As can be seen, the results have not substantially changed. The parameters estimated from this filtered dataset are very similar to those estimated from the original dataset. Thus, we conclude that the presence of potential artificially engineered genomes has not substantially impacted our analysis of natural genomes.

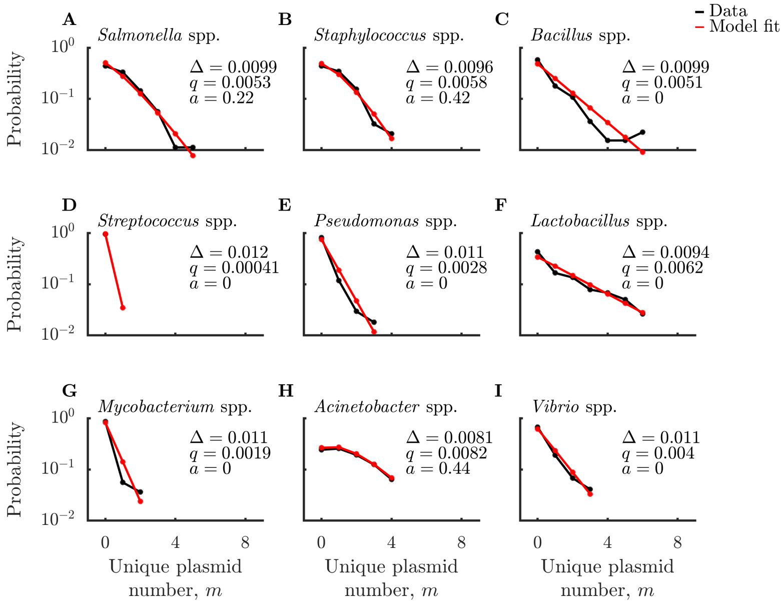

Fig. S6: Distributions of unique plasmid types per genome in common genera, along with corresponding model fits. We show the nine genera with the largest number of genomes, excluding *Klebsiella* and *Escherichia* (shown in Fig. 4B and C). We also exclude *Bordatella*, which only has one point above the observation count cutoff. Distributions truncated and fit using the same method as in Fig. 4B and C, with fitting parameters shown in each panel.

3 Multiplasmid, multipopulation Wright-Fisher models

3.1 Model formulation and stationary distribution

We begin with a Wright-Fisher model with N populations living in N demes. At every time period ("epoch"), new residents of the demes are selected via a weighted random sampling (with replacement) of the previous populations. The probability of a population being selected is proportional to its fitness, with the fitness of a cell population with i plasmids being given by w_i. Each epoch, each population is invaded by a novel plasmid with probability q. As we only keep track of the number of demes occupied by population with i $(= 0, 1, 2...)$ plasmid types, every new epoch can be described as N draws from a multinomial distribution:

$$p_i = \frac{n_i w_i (1-q)}{\sum_{j=0}^{\infty} n_j w_j} \qquad i = 0, \tag{S41}$$

$$p_i = \frac{n_i w_i (1-q) + n_{i-1} w_{i-1} q}{\sum_{j=0}^{\infty} n_j w_j} \qquad i > 0. \tag{S42}$$

Where p_i is the probability of drawing a population with i plasmids, n_i is the number of populations with i plasmids. For brevity we define $T \equiv \sum_{j=0}^{\infty} n_j w_j$. In the models we analysis, $w_0 = 1$. The stationary distribution will occur when the expectation of the multinomial draws is equal to the current population such that

$$\mathbb{E}[n_i(t+1)] = Np_i(t) = n_i(t). \tag{S43}$$

For $i = 0$, applying the stationary condition yields

$$n_0 = \frac{Nn_0(1-q)}{T} \tag{S44}$$

$$\implies T = N(1-q). \tag{S45}$$

Thus for $i > 0$ the stationary condition is

$$n_i = N\left(\frac{n_i w_i(1-q) + n_{i-1}w_{i-1}q}{N(1-q)}\right). \tag{S46}$$

For brevity we define $\kappa \equiv q/(1-q)$ and divide both sides by N to obtain an expression for $i > 0$ in terms of the population fractions, f_i:

$$f_i = f_i w_i + \kappa f_{i-1} w_{i-1}. \tag{S47}$$

This is an infinite set of equations, but we can infer the relationship between the f_0 and all later entries by examining the first few equations. We first write out the equations for $k = 1, 2, 3$:

$$f_1 = f_1 w_1 + \kappa f_0 w_0, \tag{S48}$$

$$f_2 = f_2 w_2 + \kappa f_1 w_1, \tag{S49}$$

$$f_3 = f_3 w_3 + \kappa f_2 w_2. \tag{S50}$$

Solving these equations gives a recursion relationship for $i > 0$:

$$f_i = \left(\frac{\kappa w_{i-1}}{1 - w_i}\right) f_{i-1}. \tag{S51}$$

Therefore, the expression in terms of f_0 is

$$f_i = f_0 \prod_{j=1}^{i} \left(\frac{\kappa w_{j-1}}{1 - w_j}\right) \qquad i > 0. \tag{S52}$$

For the fractions to be proper, they must all sum to 1:

$$f_0 + \sum_{i=1}^{\infty} f_0 \prod_{j=1}^{i} \left(\frac{\kappa w_{j-1}}{1 - w_j}\right) = 1. \tag{S53}$$

From this, we can solve for the value of f_0:

$$f_0 = \frac{1}{1 + \sum_{i=1}^{\infty} \prod_{j=1}^{i} \frac{\kappa w_{j-1}}{1 - w_j}}. \tag{S54}$$

Thus for $i > 0$, the population fractions are

$$f_i = \frac{\prod_{j=1}^{i} \frac{\kappa w_{j-1}}{1 - w_j}}{1 + \sum_{j=1}^{\infty} \prod_{k=1}^{j} \frac{\kappa w_{k-1}}{1 - w_k}}. \tag{S55}$$

3.1.1 Case of positive epistasis

In this version of the Wright-Fisher model, we take the plasmid-free populations to have a fitness of unity, while all plasmid-containing populations have a fitness of $1 - \Delta$. This is an extreme form of positive epistasis among plasmid types (i.e. the presence of one plasmid reduces the fitness burden of another plasmid). Every new "epoch" can again be described as N draws from a multinomial distribution:

$$p_i = \frac{n_k(1-q)}{\sum_{j=0}^{\infty} n_j (1-\Delta)^{\mathbb{1}_{j \neq 0}}} \qquad i = 0, \tag{S56}$$

$$p_i = \frac{n_i(1-\Delta)(1-q) + n_{i-1}(1-\Delta)^{\mathbb{1}_{i-1 \neq 0}} q}{\sum_{j=0}^{\infty} n_j (1-\Delta)^{\mathbb{1}_{j \neq 0}}} \qquad i > 0, \tag{S57}$$

where p_i is probability of drawing a species with i plasmids, n_i is the population of species with i plasmids, and $\mathbb{1}$ is the indicator function. For brevity we define $T \equiv \sum_{j=0}^{\infty} n_j (1-\Delta)^{\mathbb{1}_{j \neq 0}}$. The stationary distribution

will occur when the expectation of the multinomial draws is equal to the current population:

$$\mathbb{E}[n_i(t+1)] = Np_i(t) = n_i(t). \tag{S58}$$

For $i = 0$, applying the stationary condition yields

$$n_0 = \frac{Nn_0(1-q)}{T} \tag{S59}$$

$$\implies T = N(1-q). \tag{S60}$$

Thus for $i > 0$ the stationary condition is

$$n_i = N\left(\frac{n_i(1-\Delta)(1-q) + n_{i-1}(1-\Delta)^{\mathbb{1}_{i-1\neq 0}}q}{N(1-q)}\right). \tag{S61}$$

For brevity we define $\kappa \equiv q/(1-q)$ and divide both sides by N to obtain an expression for $i > 0$ in terms of the population fractions, f_i:

$$f_i = f_i(1-\Delta) + \kappa f_{i-1}(1-\Delta)^{\mathbb{1}_{i-1\neq 0}}. \tag{S62}$$

Once again we will examine the first few equations to extract a recursion relationship between population fractions. Writing out the equations for $k = 1, 2, 3$ yields:

$$f_1 = f_1(1-\Delta) + \kappa f_0, \tag{S63}$$

$$f_2 = f_2(1-\Delta) + \kappa f_1(1-\Delta), \tag{S64}$$

$$f_3 = f_3(1-\Delta) + \kappa f_2(1-\Delta). \tag{S65}$$

Solving each of these gives:

$$f_1 = \left(\frac{\kappa}{\Delta}\right) f_0, \tag{S66}$$
$$f_2 = \left(\frac{\kappa(1-\Delta)}{\Delta}\right) f_1, \tag{S67}$$
$$f_3 = \left(\frac{\kappa(1-\Delta)}{\Delta}\right) f_2. \tag{S68}$$

We can therefore extract a recurrence equation relating f_i to f_0 for $i > 0$:

$$f_i = \left(\frac{\kappa}{\Delta}\right)\left(\frac{\kappa(1-\Delta)}{\Delta}\right)^{i-1} f_0. \tag{S69}$$

Since these proportions must sum to unity, we can find f_0 from the infinite sum of the coefficients:

$$\frac{1}{f_0} = 1 + \sum_{i=1}^{\infty}\left(\frac{\kappa}{\Delta}\right)\left(\frac{\kappa(1-\Delta)}{\Delta}\right)^{i-1} = \frac{\Delta}{\Delta - q}. \tag{S70}$$

For the stationary distribution to exist this series must converge, requiring that

$$\left(\frac{\kappa(1-\Delta)}{\Delta}\right) < 1 \tag{S71}$$
$$\implies q < \Delta. \tag{S72}$$

This makes intuitive sense: in order for the number of plasmid types per cell not to increase without bound, the reduction in reproduction probability due to the presence of plasmids must be greater than the invasion probability. The entire distribution is therefore

$$f_0 = \frac{\Delta - q}{\Delta} \qquad i = 0 \tag{S73}$$
$$f_i = \left(\frac{q(\Delta - q)}{\Delta^2(1-q)}\right)\left(\frac{q(1-\Delta)}{\Delta(1-q)}\right)^{i-1} \qquad i > 0. \tag{S74}$$

Note that if $\Delta \ll 1$ and $q \ll 1$, this distribution can be reduced to a single parameter distribution:

$$f_i = \left(1 - \frac{q}{\Delta}\right)\left(\frac{q}{\Delta}\right)^{i}. \tag{S75}$$

3.1.2 Case of arbitrary invasion and fitness costs

In order to determine what classes of Wright-Fisher models are able to capture the exponential distribution seen in the data, we expand the model to include arbitrary plasmid invasion rates and fitness costs. A population with i plasmids has an invasion rate of $q(i)$ and a fitness cost of $\Delta_{\text{tot}}(i)$. The resulting multinomial is therefore

$$p_i = \frac{n_i(1-\Delta_{\text{tot}}(i))(1-q(i))}{\sum_{j=0}^{\infty} n_j(1-\Delta_{\text{tot}}(j))} \qquad i = 0, \tag{S76}$$

$$p_i = \frac{n_i(1-\Delta_{\text{tot}}(i))(1-q(i)) + n_{i-1}(1-\Delta_{\text{tot}}(i-1))q(i-1)}{\sum_{j=0}^{\infty} n_j(1-\Delta_{\text{tot}}(j))} \qquad i > 0. \tag{S77}$$

As in the previous cases, we can then apply the steady-state condition for $i = 0$ to obtain $T = N(1 - q(0))$. This gives us a general recursion relationship for $i > 0$:

$$f_i = \frac{f_i(1-\Delta_{\text{tot}}(i))(1-q(i)) + f_{i-1}(1-\Delta_{\text{tot}}(i-1))q(i-1)}{1-q(0)}. \tag{S78}$$

Note that here we assume $\Delta(0) = 0$. We can rearrange this to obtain an expression for the ratio of consecutive entries of the distribution

$$\frac{f_i}{f_{i-1}} = \frac{(1-\Delta_{\text{tot}}(i-1))q(i-1)}{1-q(0)-(1-\Delta_{\text{tot}}(i))(1-q(i))}, \tag{S79}$$

$$\frac{f_i}{f_{i-1}} = \frac{q(i-1)-\Delta_{\text{tot}}(i-1)q(i-1)}{1-q(0)-1+\Delta_{\text{tot}}(i)+q(i)-\Delta_{\text{tot}}(i)q(i).} \tag{S80}$$

In limit of small invasion probability and fitness cost, this reduces to:

$$\frac{f_i}{f_{i-1}} = \frac{q(i-1)}{q(i)-q(0)+\Delta_{\text{tot}}(i)} = K_i. \tag{S81}$$

In order to yield an exponential distribution, $K_i = K < 1 \; \forall i$. Imposing this condition leads to:

$$\frac{q(i-1)}{K} - q(i) + q(0) = \Delta_{\text{tot}}(i). \tag{S82}$$

This result indicates that there are multiple models that can produce an exponential distribution. For example, if $q(i) = q_{\text{p}} + q_{\text{p}} i$ and $\Delta_{\text{tot}}(i) = \Delta_{\text{p}} i$, an exponential distribution can be recovered if $\Delta_{\text{p}} = \left(\frac{1}{K} - 1\right) q_{\text{p}}$.

3.1.3 Incompatibility interactions

How would our model behave if we included incompatibility interactions between plasmids? First, let us consider cells sampling from a uniform distribution of plasmids. Suppose there are N distinct, equally abundant, incompatibility groups that can coexist over long timescales within a single cell. Whenever a plasmid invades a cell, its incompatibility group is randomly selected from these incompatibility groups, and the invasion is only successful if the incoming plasmid belongs to a new incompatibility group. In this case, we can represent this with an invasion function $q = q_0(N - i)/N$ where i is the number of plasmids in the cell and q_0 is the invasion probability for a cell containing no plasmids. The outcome will depend on the number of incompatibility groups. If the number of plasmids contained within the average cell is small relative to the number of incompatibility groups, the outcome will be similar to that of a constant invasion probability. This is because the invasion probability does not change substantially if $i \ll N$. However, if there are a small number of incompatibility groups, this would have an effect similar to negative fitness epistasis and shorten the tail of the plasmid distribution. Any deviation from a uniform distribution of plasmids would actually shorten the tail of the plasmid distribution. This is because it would be more likely for plasmids that already reside within the cell to attempt and fail to invade, effectively reducing the invasion rate and thus favoring lower plasmid numbers.
